# Dental Sealant Empowered by 1,3,5-Tri Acryloyl Hexahydro-1,3,5-Triazine and α-Tricalcium Phosphate for Anti-Caries Application

**DOI:** 10.3390/polym12040895

**Published:** 2020-04-12

**Authors:** Juliana Caletti Monteiro, Michele Stürmer, Isadora Martini Garcia, Mary Anne Melo, Salvatore Sauro, Vicente Castelo Branco Leitune, Fabrício Mezzomo Collares

**Affiliations:** 1 Dental Materials Laboratory, School of Dentistry, Federal University of Rio Grande do Sul, Rua Ramiro Barcelos, 2492, Rio Branco, Porto Alegre RS 90035-003, Brazil; juliana.caletti@hotmail.com (J.C.M.); mi_sturmer@hotmail.com (M.S.); isadora.garcia@ufrgs.br (I.M.G.); vicente.leitune@ufrgs.br (V.C.B.L.); 2 Ph.D. Program in Biomedical Sciences, University of Maryland School of Dentistry, Baltimore, MD 21201, USA; MMelo@umaryland.edu; 3 Operative Dentistry Division, General Dentistry Department University of Maryland School of Dentistry, Baltimore, MD 21201, USA; 4 Departamento de Odontología, Facultad de Ciencias de la Salud, Universidad CEU-Cardenal Herrera, C/Del Pozo (s/n), Alfara del Patriarca, 46115 Valencia, Spain; salvatore.sauro@uchceu.es; 5 Department of Therapeutic Dentistry, Sechenov University of Moscow, Mozhaisky Val, 11, Moscow 119435, Russia

**Keywords:** dental resin, antibacterial, polymerization, remineralizing, triazines

## Abstract

Quaternary ammonium compounds and calcium phosphates have been incorporated into dental materials to enhance their biointeractivity and preventive effects. This study aimed at evaluating the physical and chemical properties and effects against *Streptococcus mutans* of a dental sealant containing 1,3,5-tri acryloyl hexahydro-1,3,5-triazine (TAT) and α-tricalcium phosphate (α-TCP). A methacrylate-based dental sealant was initially formulated. α-TCP and TAT (G_α-TCPTAT_) were added to the experimental sealant at 2 wt.% each. One group was formulated without α-TCP and TAT and used as control (G_CTRL_). All tested resins were analyzed for polymerization kinetics and degree of conversion (DC %), Knoop hardness (KHN), softening in solvent (∆KHN%), ultimate tensile strength (UTS), the contact angle with water or with α-bromonaphthalene, surface free energy (SFE) and antibacterial activity against *Streptococcus mutans* in biofilm and in planktonic cells. The polymerization kinetic was different between groups, but without statistical differences in the DC % (*p* < 0.05). KHN and ΔKHN% did not change between groups (*p* > 0.05), but G_α-TCPTAT_ presented greater UTS compared to G_CTRL_ (*p* < 0.05). No differences were found for contact angle (*p* > 0.05) or SFE (*p* > 0.05). G_α-TCPTAT_ showed greater antibacterial activity in comparison to G_CTRL_ (*p* < 0.05). The formulation of dental sealants containing TAT and α-TCP can be characterized by improved mechanical and antibacterial properties.

## 1. Introduction

Dental caries is the foremost oral health problem around the world and it is one of the most common conditions affecting children [1]. *Streptococcus mutans* (*S. mutans*) presents a major role within the pathogenic biofilm that triggers dental caries. S. mutans adheres to the tooth surface and metabolizes carbohydrates, producing acids that lead to enamel demineralization for the onset of dental caries, as well its progression. Although dental caries is a multifactorial disease, it can be prevented and even potentially reversed during its early stages [2]. Indeed, resin sealants for pits and fissures have been used in order to reduce the incidence of new caries lesions in high-risk patients, as well as to treat non-cavitated lesions [3]. The effectiveness of sealants mainly depends on their retention and sealing; there is strong evidence that the rate of remained intact resin decreases over time [4], leading to the need for the development of novel materials with better mechanical properties. Furthermore, the current trend in material science involves the incorporation of copolymerizable antimicrobial agents and bioactive remineralizing fillers [5].

1,3,5-tri acryloyl hexahydro-1,3,5-triazine (TAT) is a quaternary ammonium compound (QAC) showing selective antibacterial properties against gram-positive pathogens [6], such as S. mutans. This compound has already been used in dental materials formulations, such as dentin bonding agents, composite resins and orthodontic adhesives [7,8,9]. It is a current trend to improve the therapeutics properties of dental materials by incorporating releasing or non-releasing antibacterial agents within their formulations. The disadvantage of using an antimicrobial agent that releases over time is the loss of activity [10]. Due to its tri-functional molecule - with three methacrylate groups – TAT has the ability to copolymerize with the methacrylate resin matrix and holds more extended antibacterial activity [7]. Furthermore, materials with copolymerized TAT seem to be characterized by better physical and chemical properties [9].

Furthermore, calcium phosphates have been incorporated in dental materials to induce the release of calcium and phosphates ions in order to promote mineral deposition and dental remineralization [11,12,13]. One of our previous studies showed that α-tricalcium phosphate (α-TCP) at 2 wt.% in an adhesive resin was able to promote mineral deposition on dentin after selective removal of carious tissue, besides improving the bond strength and show better results among other calcium phosphates [12]. However, there is no information about the combination of TAT and α-TCP, aiming to provide biointeractivity for dental materials. Considering that dental sealants could be improved, we decided to investigate the combined antibacterial and bioactive agents already tested by our research group for the development of a low viscosity dental resin intended for preventing the caries onset. This study aimed at evaluating the physical, chemical and antibacterial properties against *Streptococcus mutans* of an experimental dental sealant containing 1,3,5-tri acryloyl hexahydro-1,3,5-triazine (TAT) and α-tricalcium phosphate (α-TCP).

## 2. Materials and Methods

### 2.1. Resin Sealants Formulation

**Figure 1:**
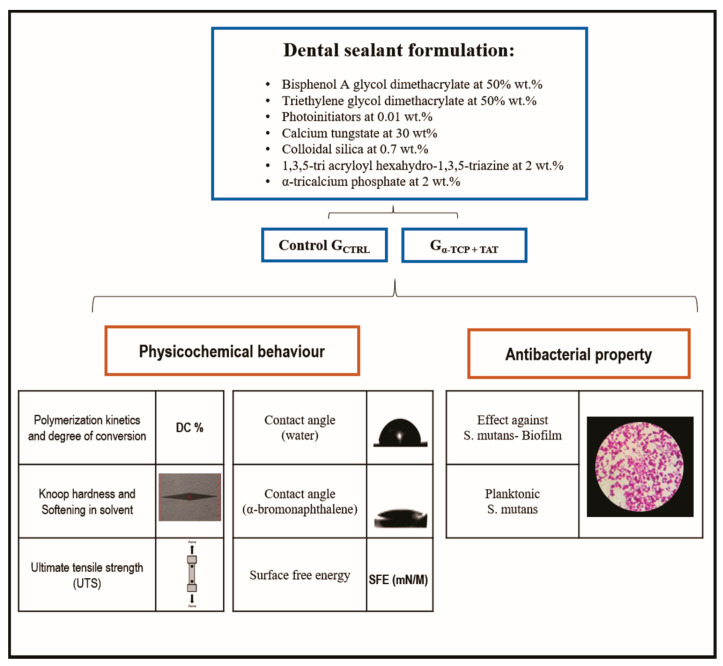
Schematic draw of the study design.

For resin sealants formulation, bisphenol A glycol dimethacrylate (BisGMA) at 50 wt.% and triethylene glycol dimethacrylate (TEGDMA) at 50 wt.% were used. Camphorquinone (CQ) and ethyl 4-dimethylaminobenzoate (EDAB) at 1 mol%, according to the monomer moles were added as a photoinitiator system. Butylated hydroxytoluene (BHT) at 0.01 wt.% was also used as a stabilizer. Calcium Tungstate was added as a radiopacifier agent at 30 wt.%. Colloidal silica was added at 0.7 wt.% for viscosity adjustment, while α-TCP [12] and TAT (G_α-TCPTAT_) were added to the experimental resin sealant at 2 wt.% each. A control resin sealant was formulated without the use of α-TCP and TAT (G_CTRL_). All the chemicals listed so far were purchased from Aldrich Chemical Company, St. Louis, MI, USA. All specimens, except those for ultimate tensile strength evaluation, were prepared using a polyvinylsiloxane mold with 1 mm thickness and 4 mm diameter. Next, the resin was photoactivated for 30 s on each side using a light-curing unit (Radii Cal, SDI, Bayswater, Victoria, Australia) at 1200 mW/cm^2^. The specimens for the polymerization kinetics were photoactivated for 30 s on the top. Figure 1 displays a schematic diagram of the experimental design and the type of assessments employed in this study.

### 2.2. Polymerization Kinetics and Degree of Conversion (DC %)

The polymerization kinetics and the DC % of the experimental resin sealants were evaluated through Fourier transform infrared spectroscopy (FTIR, Bruker Optics, Ettlingen, Baden-Württemberg, Germany). Drops of the unpolymerized sealants were dispensed on the attenuated total reflectance device (ATR). Three specimens per group were photoactivated at 1 mm between the tip of the light-curing unit and the top of each specimen. During the photoactivation, two spectra per second were obtained in absorbance mode (10 kHz velocity, 4 cm^−1^ resolution, Opus 6.5 software, Bruker Optics, Ettlingen, Baden-Württemberg, Germany) from 4000 to 400 cm^−1^. DC % was calculated using the first spectrum of the uncured resin and the last spectrum, when the resin was totally polymerized. The DC % was calculated using the peak at 1610 cm^−1^ from aromatic carbon-carbon double bond as the internal standard, along with the peak at 1640 cm^−1^ as an aliphatic carbon-carbon double bond [14]. The following formula was applied:

$$\mathrm{DC}\;\%=100\times \left(\frac{\mathrm{peak}\;\mathrm{height}\;\mathrm{of}\;\mathrm{cured}\;\mathrm{aliphatic}\;\mathrm{C=C}/\mathrm{peak}\;\mathrm{height}\;\mathrm{of}\;\mathrm{cured}\;\mathrm{aromatic}\;\mathrm{C=C}}{\mathrm{peak}\;\mathrm{height}\;\mathrm{of}\;\mathrm{uncured}\;\mathrm{aliphatic}\;\mathrm{C=C}/\mathrm{peak}\;\mathrm{height}\;\mathrm{of}\;\mathrm{aromatic}\;\mathrm{C=C}}\right).$$

The polymerization rate (Rp) was calculated by subtracting the DC % at a time “t” from the DC achieved at time “t−1.” The graphs of DC % versus time, Rp versus time and DC % versus Rp were plotted.

### 2.3. Knoop Hardness and Softening in Solvent

Five specimens were prepared and embedded in self-curing acrylic resin and polished in an automatic polisher machine (Model 3v, Arotec, Cotia, São Paulo, Brazil) using silicon carbide sandpapers with decreasing grain size up to 2000 grit and under continuous irrigation using distilled water. Felt discs with alumina suspension (0.5 µm, Arotec, Cotia, São Paulo, Brazil) were used for the final polishing step. After 24 h, five indentations (10 g/5s) were performed on the top of each specimen using a digital microhardness tester (HMV 2, Shimadzu, Tokyo, Honshu, Japan) to obtain the initial Knoop hardness number (KHN1). Each group was immersed in a Becker flask with a solution of ethanol: water (70:30) for two hours and washed with distilled water for subsequent evaluation of the final Knoop hardness number (KHN2). The difference between KHN2 and KHN1 is presented as the percentual Knoop hardness reduction (ΔKHN%).

### 2.4. Ultimate Tensile Strength (UTS)

Ten specimens per group (8.0 mm long, 2.0 mm wide and 1.0 mm thickness) were prepared using a metallic matrix with a cross-sectional area of ±1 mm^2^ (±0.1 mm^2^) and an hourglass shape. After 24 h, the samples were glued onto metallic jigs using the cyanoacrylate resin. A micro tensile strength test was performed using a universal testing machine (EZ-SX Series, Shimadzu, Kyoto, Japan). The specimens were stressed at 1 mm/min crosshead speed until fracture. The result in MPa was calculated by dividing the maximum force value to the fracture (N) by the area of each sample at the constriction at the hourglass shape (mm²).

### 2.5. Contact Angle and Surface Free Energy (SFE)

The contact angle and the surface free energy (SFE) of the experimental resin sealants were evaluated using five specimens per group. First, specimens were embedded in acrylic resin and then polished, as described in the softening in the solvent section. An optical tensiometer (Theta, Biolin Scientific, Stockholm, Stockholm, Sweden) was used with the sessile drop method with one drop of distilled water (used as a polar liquid) or α-bromonaphthalene (used as a non-polar liquid) that was dispensed on the polymerized specimens. The drop-out size was 3.0 μL, the drop rate was 2.0 μL/s, the displacement rate was 20.0 μL/s and the speed dispersion of water or α-bromonaphthalene was 50 mm/min. The drop was evaluated on the polymerized specimens during 20 s and the mean contact angle was registered after 10 s after liquid contact with the surface. The SFE was obtained with the Owens-Wendt-Rabel-Kaelble (OWRK) method [15] in the OneAttension software (Biolin Scientific, Stockholm, Stockholm, Sweden).

### 2.6. Activity against Streptococcus Mutans

The antibacterial activity was evaluated for biofilm and planktonic bacteria against *Streptococcus mutans* (NCTC 10449). Three specimens per group were prepared for the biofilm test and three samples for the planktonic bacteria evaluation. Firstly, bacteria were prepared for the tests: 300 µL of frozen *S. mutans* in skim milk was placed on a Petri dish containing brain heart infusion (BHI) broth with agar at 15 g/L and kept in an oven, in a microaerophilic environment with 5 % of CO_2_, at 37 ºC for 48 h. The colonies were collected, placed in BHI broth containing 1 wt.% of sucrose and kept at 37 °C for 24 h in the microaerophilic environment. The initial inoculum used for the tests was assessed by taking 100 µL of the subcultured broth and mixing it with 900 µL of a sterile saline solution (0.9%) in an Eppendorf tube. A serial dilution was performed by vortexing the first to the sixth Eppendorf tube to dilute the solution until 10^−6^ mL. The solutions were plated on BHI agar (two drops of 25 µL) and kept under the microaerophilic environment at 37 °C for 48 h. The colonies were visually counted and transformed in colony-forming units per milliliter (CFU/mL), indicating an inoculum used at 5 × 10^6^ CFU/mL.

Subsequently, the antibacterial activity against biofilm formation on the polymerized specimens was evaluated. The specimens were fixed on the lid of a 48-well plate and sterilized using hydrogen peroxide plasma (58%) for 48 min at 56 °C. In the sterile 48-well plate, 100 μL of the inoculum previously grown was inserted in each well with 900 μL of BHI broth containing 1 wt.% of sucrose. The lid and the specimens were combined with the 48-well plate base filled with the broth containing the bacteria and kept under 37 °C for 24 h to form the biofilm on the top of the samples. Subsequently, the specimens were removed from the lid and placed in an Eppendorf with 1 mL of saline solution to be vortexed for 1 min and serially diluted, as previously stated. The diluted solutions were placed in Petri dishes with BHI agar to count the CFU/mL, as mentioned above and expressed in log CFU/mL.

The broth with bacteria that was in contact with the samples for 24 h was used to evaluate the antibacterial activity against planktonic bacteria. From each well, 100 µL were collected and inserted in Eppendorf tube with 900 µL of saline solution to be vortexed, diluted until 10^−6^ and platted in BHI agar Petri dishes. Broth and *S. mutans* in three wells without samples’ contact were used as a negative control. CFU/mL was counted and expressed in log CFU/mL.

### 2.7. Statistical Analysis

The data were analyzed using the software SigmaPlot, version 12.0 (Systat Software, Inc., San Jose, CA, USA). Data distribution was evaluated by the Shapiro-Wilk test. Paired t-test was used to compare KHN1 and KHN2 in each group. Student t-test was used to compare groups in all tests at a level of 0.05 of significance.

## 3. Results

The values of degree of conversion (DC %), initial and final Knoop hardness (KHN1, KHN2), softening in solvent (ΔKHN%) and ultimate tensile strength (UTS) of the experimental resin sealants are depicted in Table 1. It was possible to observe that the DC % ranged from 49.67 (±4.47) % to 51.08 (±3.17) % without significant differences between the groups (*p* > 0.05). There was no significant difference for KHN1 (*p* > 0.05); all groups presented a decrease in the Knoop hardness after two hours of immersion in the ethanol solution (*p* < 0.05). The ΔKHN% ranged from 62.64 (±4.98) % to 65.02 (±4.83) %, but no significant difference was encountered between the groups (*p* > 0.05). When TAT and α-TCP were incorporated into the experimental dental sealant, the mechanical property evaluated through UTS showed higher values in comparison to G_CTRL_ (*p* < 0.05).

**Figure 2:**
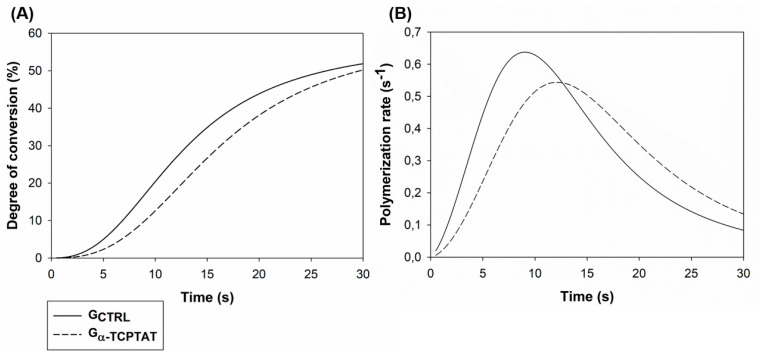
Polymerization behavior evaluation for 30 s of the experimental resin sealants. (**A**) DC % versus photoactivation time. (**B**) Rp versus photoactivation time.

Figure 2 shows the polymerization behavior of the experimental dental sealants. The DC % during time showed a slightly higher value of DC % for G_CTRL_. The Rp versus time graph shows a delay in the polymerization reaction for G_α-TCPTAT_ in comparison to G_CTRL_. However, there was no significant difference for the conversion of carbon-carbon double bonds of the tested materials after 30 s of photoactivation, indicating that the addition of TAT and α-TCP did not modify the DC % of the sealant.

The values generated during the evaluation of the contact angle with water or α-bromonaphthalene and SFE of the experimental dental sealants are presented in Table 2. The contact angle with water or α-bromonaphthalene was not different between the tested groups (*p* > 0.05). The SFE ranged between 48.68 (±1.40) mN/M and 49.08 (±6.05) mN/M, with no significant statistical difference between G_α-TCPTAT_ and G_CTRL_ (*p* > 0.05).

Table 3 displays the antibacterial effect against *Streptococcus mutans*. G_CTRL_ presented 6.38 (±0.57) log CFU/mL, while G_α-TCPTAT_ showed 4.95 (±0.30) log CFU/mL. G_α-TCPTAT_ significantly reduced bacterial growth on the surface of the polymerized sealants in comparison to G_CTRL_ (*p* < 0.05). G_α-TCPTAT_ also showed a significant reduction of planktonic bacteria viability when compared to G_CTRL_ and negative control (*p* < 0.05).

## 4. Discussion

**Figure 3:**
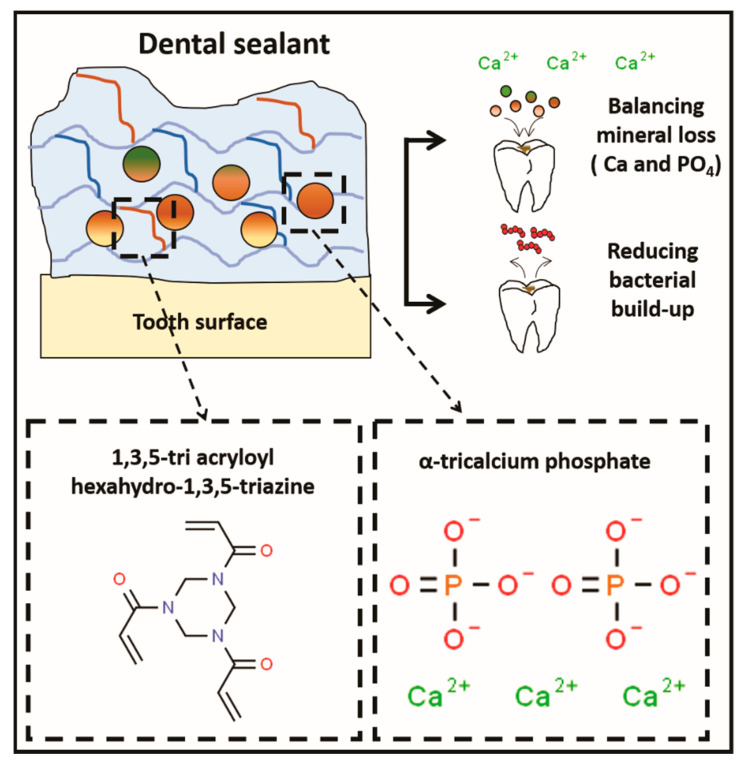
Schematic draw of the dental sealant formulated and analyzed in the present research, containing an antibacterial and a bioactive agent.

Resin-based sealants are effective in preventing and reducing caries because they work as a physical barrier that prevents tooth demineralization [3]. The antibacterial monomer TAT and α-TCP, a remineralizing agent (Figure 3), were incorporated at 2 wt.% each in an experimental resin sealant. G_α-TCPTAT_ showed antibacterial activity against biofilm formation and planktonic cells of *S. mutans* and increased the UTS values without compromising the physicochemical properties of the sealant.

All the experimental resin sealants were formulated and evaluated regarding the polymerization kinetics and DC % by FTIR-ATR. DC % represents the percentage of carbon double bond conversion and high values are associated with better mechanical properties and reduced matrix degradation [16]. TAT is a tri-functional monomer, presenting three aliphatic double bonds (C=C) that are able to copolymerize, which may also increase double bond conversion [9]. In the polymerization kinetics regarding DC % per time, no significant statistical difference was observed between the tested groups. On the other hand, at the polymerization rate per time analysis, the difference can be observed in Figure 1, indicating that the reaction was delayed for G_α-TCPTAT_. Such a result may be related to an increase of resin sealant viscosity, as well as to the decrease of monomers mobility for G_α-TCPTAT_, leading to a non-difference of DC % between groups even with the addition of this trifunctional molecule (TAT) [13]. G_α-TCPTAT_ presents more inorganic filler due to α-TCP addition, which may have caused a reduction of the transmission of the light during the light-curing procedures, so reducing the DC % compared to G_CTRL_ [17]. The presence of TAT may have made it possible to incorporate a filler (α-TCP) with a very different refractive index than the resin matrix without changing the polymerization degree. Nevertheless, both groups achieved reliable DC %, with values comparable to those of resin sealants currently available in the dental market [18]. 

TAT has been used to synthesize materials with better chemical and physical properties [7,8]. In the current study, the Knoop hardness and the softening in solvent, which can be related to polymers’ crosslinking density, showed no differences between groups. A lower concentration of TAT was used than the previous studies [9], which could lead to no detectable differences in Knoop hardness. This test evaluates a superficial characteristic of the material and it can change polymers’ values due to the load pressure on inorganic fillers (such as calcium tungstate and calcium phosphate) in the softer organic matrix. Thus, it may not measure the intrinsic value of the crosslinked resin [19] due to the presence of more amount of fillers.

Conversely, the mechanical properties (ultimate tensile strength) of G_α-TCPTAT_ were greater than those observed in the G_CTRL_. This mechanical improvement may be related to higher crosslinking density with TAT, although no significant difference was found for DC % or Knoop hardness. The presence of higher filler content (α-TCP) can increase the mechanical properties of composite materials [20], such as the experimental dental sealant tested in this study. However, agglomerated inorganic fillers within the polymer could also increase internal defects in the materials, leading to reduced mechanical properties [21]. The results of UTS may indicate the homogeneous dispersion of filler particles in the groups containing α-TCP and TAT. These were promising results since it is desired that resin sealants present reliable mechanical properties to offer suitable resistance when receiving loads in occlusal dental surfaces during masticatory forces. It is well known that mechanical tests under static or dynamic loading can induce different outcomes for materials’ behavior [22]. With the promising results observed for the composite with TAT here formulated, further studies may be performed to analyze its mechanical properties considering different designs.

The incorporation of fillers in polymers may also change the contact angle and surface free energy (SFE) [23]. Besides, a previous study showed that the penetration coefficient (PC) of commercial sealants is correlated to their ability to penetrate fissures. The PC equation was composed of the liquid’s surface tension to air, the cosine of the liquid’s contact angle to enamel and the dynamic viscosity of the fluid. Therefore, at low contact angle values, a high sealant PC can be achieved [24]. G_αTCPTAT_ had no significant influence on SFE and contact angle, which probably lead to an increased PC.

QACs have been studied due to their high and broad antibacterial spectra, even at small concentrations [25,26,27]. In the case of TAT, it was suggested that the mechanism of antibacterial action is due to “contact killing” effect [6]. QACs are positively charged due to a lack of electrons in the nitrogen atom, which provides an electrostatic interaction with the negatively charged bacteria’s membrane/wall. This event possibly leads to a disruption of bacteria membrane and loss of intracellular components, as previously shown for other QACs [28]. In this study, G_α-TCPTAT_ reduced bacterial growth on the surface of the polymerized samples compared to G_CTRL_, corroborating to previous analyses of TAT in resins [7,8,9]. G_α-TCPTAT_ also reduced the number of planktonic bacteria, similar to other studies with copolymerized QAC [29]. It is a limitation of this study that the sealant was not tested against a complex multi-species biofilm, which would better mimic a clinical situation [30]. However, here we tested the effect of a novel sealant against a bacterium positively correlated with dental caries (S. mutans) [31] and the results encourage further evaluation in situ and in vivo.

## 5. Conclusions

Dental sealants empowered by 1,3,5-tri acryloyl hexahydro-1,3,5-triazine and α-tricalcium phosphate for anti-caries application presented lower bacterial growth and tensile strength. Moreover, such an experimental material formulated with antibacterial and bioactive agents showed higher resistance against the ethanol softening ageing. The combinatorial effect of bioactive and antibacterial agents might impair the developed material with dual effect—prevention and treatment of non-cavitated caries lesions on occlusal and proximal surfaces. Therefore, the innovative bioactive dental material here proposed may represent an exciting approach to contribute to the development of new anti-caries dental materials.

## Figures and Tables

**Table 1:** Mean and standard deviation values of degree of conversion (DC %), microhardness before (KHN1) and after (KHN2) the immersion in the solvent, the variation of microhardness (ΔΚHΝ%) and ultimate tensile strength (UTS).

Group	DC %	KHN1	KHN2	ΔKHN%	UTS (MPa)
G_CTRL_	51.08 (±3.17) ^A^	16.41 (±0.97) ^Aa^	6.10 (±0.53) ^b^	62.64 (±4.98) ^A^	46.71 (±3.52) ^B^
G_α-TCPTAT_	49.67 (±4.47) ^A^	18.41 (±2.22) ^Aa^	6.40 (±0.83) ^b^	65.02 (±4.83) ^A^	55.61 (±3.33) ^A^

Different capital letters indicate a statistical difference in the same column (*p* < 0.05). Different lowercase letters indicate a statistical difference in the same line for KHN1 and KHN2 of the same material (*p* < 0.05).

**Table 2:** Mean and standard deviation values of contact angle with distilled water and α-bromonaphthalene and surface free energy (SFE) of experimental sealant resins.

Group	Contact Angle (Water)	Contact Angle (α-Bromonaphthalene)	SFE (mN/M)
G_CTRL_	67.71 (±3.32) ^A^	24.69 (±3.09) ^A^	48.68 (±1.40) ^A^
G_α-TCPTAT_	66.49 (±10.9) ^A^	26.96 (±3.77) ^A^	49.08 (±6.05) ^A^

Same capital letters indicate no statistical difference in the same column (*p* > 0.05).

**Table 3:** Mean and standard deviation values of log-transformed colony-forming units/ mL (CFU/mL) for bacteria in biofilm or planktonic bacteria after the contact during 24 h with the experimental dental sealants.

Group	Effect against S. Mutans	
	Biofilm	Planktonic Bacteria
G_CTRL_	06.38 (±0.57) ^A^	09.21 (±0.14) ^A^
G_α-TCPTAT_	04.95 (±0.30) ^B^	07.73 (±0.56) ^B^
Negative control	-	09.14 (±0.10) ^A^

Different capital letters indicate a statistical difference between groups in the same column (*p* < 0.05).
